# Advances in Methanol Production and Utilization, with Particular Emphasis toward Hydrogen Generation via Membrane Reactor Technology

**DOI:** 10.3390/membranes8040098

**Published:** 2018-10-18

**Authors:** Francesco Dalena, Alessandro Senatore, Marco Basile, Sarra Knani, Angelo Basile, Adolfo Iulianelli

**Affiliations:** 1 Chemistry & Chemical Technologies Department, University of Calabria, Cubo 15/D, Via P. Bucci, 87036 Rende, CS, Italy; francesco.dalena@unical.it (F.D.); a.senatore@outlook.com (A.S.); 2 Department of Ambient, Territory and Chemical Engineering, University of Calabria, Cubo 44/A, Via P. Bucci, 87036 Rende, CS, Italy; marcobasile90@live.it; 3 Laboratoire de Chimie des Matériaux et Catalyse, Département de Chimie, Faculté des Sciences de Tunis, Université Tunis El Manar, Tunis 2092, Tunisia; sarraknani2@gmail.com; 4 Institute on Membrane Technology of the Italian National Research Council (CNR-ITM), Via P. Bucci, c/o University of Calabria, Cubo 17/C, 87036 Rende, CS, Italy; a.basile@itm.cnr.it

**Keywords:** methanol, steam reforming, water gas shift, partial oxidation, membrane reactors, hydrogen

## Abstract

Methanol is currently considered one of the most useful chemical products and is a promising building block for obtaining more complex chemical compounds, such as acetic acid, methyl tertiary butyl ether, dimethyl ether, methylamine, etc. Methanol is the simplest alcohol, appearing as a colorless liquid and with a distinctive smell, and can be produced by converting CO_2_ and H_2_, with the further benefit of significantly reducing CO_2_ emissions in the atmosphere. Indeed, methanol synthesis currently represents the second largest source of hydrogen consumption after ammonia production. Furthermore, a wide range of literature is focused on methanol utilization as a convenient energy carrier for hydrogen production via steam and autothermal reforming, partial oxidation, methanol decomposition, or methanol–water electrolysis reactions. Last but not least, methanol supply for direct methanol fuel cells is a well-established technology for power production. The aim of this work is to propose an overview on the commonly used feedstocks (natural gas, CO_2_, or char/biomass) and methanol production processes (from BASF—Badische Anilin und Soda Fabrik, to ICI—Imperial Chemical Industries process), as well as on membrane reactor technology utilization for generating high grade hydrogen from the catalytic conversion of methanol, reviewing the most updated state of the art in this field.

## 1. Introduction

In the last century, fossil fuels represented the main source of energy production. These feedstocks are not renewable, are limited and, consequently, are responsible for an instable global market, which leads to a corresponding instability in fuel price. Moreover, fossil fuel exploitation is considered primarily responsible for greenhouse gas (GHG) emissions, contributing to the increase in global warming.

Today, the most viable options for the exploitation of fossil fuels for power production result from hydrogen and methanol. The use of hydrogen appears very promising, as it shows the highest energy content per unit of weight (142 kJ/g) over any other known fuel and, furthermore, it is environmentally safe [1]. However, the key issues for wide hydrogen utilization as a new energy carrier are represented by its purification costs and by the difficulties linked to the infrastructure for its storage and transportation.

By contrast, methanol is easily stored and transported and can be used as a convenient hydrogen carrier. It is also particularly useful in the chemical industry as a solvent and as a C1 building block for producing intermediates and synthetic hydrocarbons, including polymers and single-cell proteins [2,3]. In vehicle transportation, methanol can be mixed with conventional petrol, without requiring any technical modification to the vehicle fleet. In fact, most methanol-fueled vehicles currently use M85 fuel, which represents a mixture containing 85% methanol and 15% unleaded gasoline [4].

**Figure 1:**
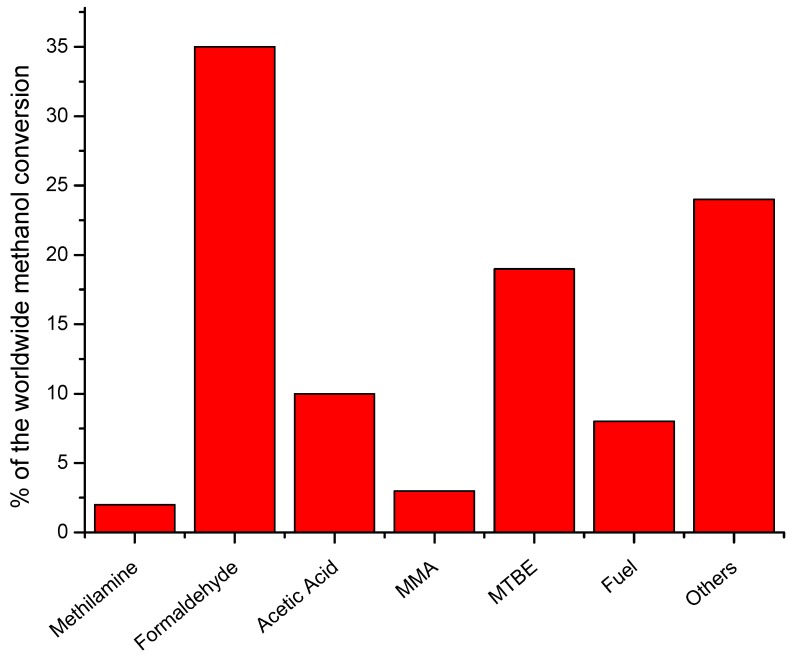
Products coming from methanol transformation.

Approximately 65% of methanol worldwide is consumed for the production of acetic acid, methyl and vinyl acetates, methyl methacrylate (MMA), methylamines, metil-t-butil etere (MTBE), fuel additives, and other chemicals. The remaining portion is converted into formaldehyde and other products [3,5,6], as illustrated in Figure 1.

A number of technologies were developed over the years to produce methanol, including several feedstocks, such as natural gas, coal, and biomass or CO_2_—the latter directly recoverable from the atmosphere [3,7].

From an historic point of view, methanol production processes took place before the 1660s (by Robert Boyle). An important contribution to its development was by Paul Sabatier, who carried out the hydrogenation of a large variety of functional groups by metal-based catalysis [8]. Methanol synthesis was performed by BASF (Germany) in 1923, developing a metal-based catalytic hydrogenation process at high pressure [9]. The BASF process has then been utilized since 1927 by both DuPont and the Commercial Solvents Corporation in the USA, representing the start point in the methanol production industry and remaining the dominant technology for over 45 years [10].

Successively, in the 1940s, the Swiss Lonza Company produced methanol industrially from electrolytic hydrogen and CO_2_, the latter derived from Ca(NO_3_)_2_ synthesis. The reactant gas purification from nitrous vapors was then developed by Natta (Italy) and combined with the methanol synthesis from CO and H_2_ [11].

The successive development of the steam methane reforming (SMR) reaction, able to generate syngas (a mixture of H_2_, CO, and CO_2_) combined with active Cu/ZnO catalysts, made it possible to operate at milder conditions, such as 300 °C and 100 bar. This was the core of the “ICI process”, proposed in 1966 [3].

In 1973, during the oil embargo proclaimed by the Organization of Arab Petroleum Exporting Countries in the USA and The Netherlands, the interest toward methanol exploitation as an alternative automobile fuel was particularly intense, even though it was definitively banned after the 1990s due to the harmful methanol combustion products negatively affecting the ozone layer. In the same period, methanol market demand grew once again as a consequence of its large utilization as a fertilizer in agriculture.

**Figure 2:**
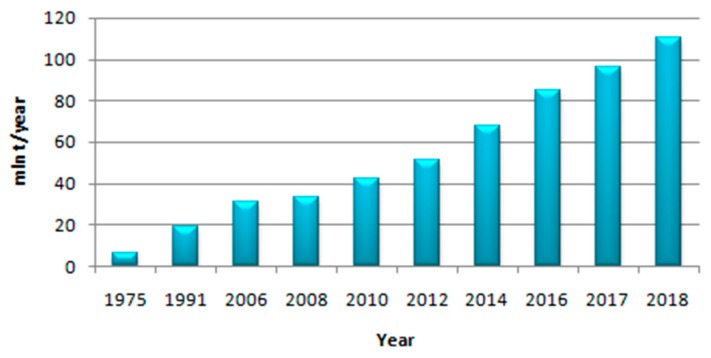
Methanol production expressed in Mt per year of production from 1975 until 2018 (October).

Methanol is currently produced all around the world (over 90 methanol plants facilitate a production capacity of around 110 million tons [3], Figure 2): North and South America, Europe, Asia, and there are also some plants in Africa. Moreover, global methanol consumption is valued at an average of around 100 Mt/year, growing very fast in the last four decades [7,12].

In the last three decades, the potential of methanol utilization as a hydrogen carrier was also demonstrated [13]. Furthermore, an extensive range of literature was addressed at hydrogen production through steam reforming reaction [14,15,16,17,18,19,20]. Compared to steam methane reforming (SMR) reaction (usually carried out between 800 °C and 1000 °C), methanol steam reforming (MSR) attracted particular interest because it takes place at significantly lower temperatures, around 240–260 °C. The MSR process can be described by three partial chemical reactions:

CH_3_OH + H_2_O = CO_2_ + 3H_2_   ∆H°_298K_ = +49.7 (kJ/mol), (1)

CO + H_2_O = CO_2_ + H_2_   ∆H°_298K_ = −41.2 (kJ/mol), (2)

CH_3_OH = CO + 2H_2_   ∆H°_298K_ = +90.7 (kJ/mol). (3)

The MSR reaction is represented by Equation (1), and Equation (2) is the water gas shift (WGS) reaction, while Equation (3) is the methanol decomposition reaction. Apart from in Equations (1) and (3), which are endothermic and involve a volume increase, the WGS reaction is exothermic and occurs without volume change.

In the following, the state of the art about methanol production, its representative production processes, the most interesting advancements on its utilization for generating hydrogen, combined to the application of methanol in fuel cells is reported.

## 2. Methanol Production

Methanol is producible from a number of carbon-based feedstocks, such as natural gas, coal, biomass, and CO_2_. Successively, some of the most important methanol production processes are hence examined in detail and related reactions and technologies discussed in depth.

### 2.1. Natural Gas as a Primary Source of Methanol Production

Today, around 90% of methanol comes from natural gas [21]. The steps for producing methanol are relatively straightforward and can be summarized to the followings three basic stages: (1) Synthesis gas production; (2) syngas conversion into crude methanol; (3) crude methanol distillation to reach the required purity [22,23,24].

Both steam and autothermal reforming (SMR and AMR, respectively) of natural gas (Equations (4) and (5)) are needed to generate the syngas mixture (mainly composed by H_2_, CO, and CO_2_), even though it can also be obtained via partial oxidation of methane (POM) (Equation (6)) or different carbon-based materials, such as coal, heavy oils, or biogases [25].

CH_4_ + H_2_O = CO + 3H_2_ (4)

CH_4_ + 2O_2_ → CO_2_ + 2H_2_O (5)

CH_4_ + ½O_2_ → CO + 2H_2_ (6)

The composition of syngas normally depends on the S ratio (Equation (7)), represented by the hydrogen and carbon dioxide moles difference over the CO_2_ and CO moles sum.

(7) $$S=\;\frac{{\mathrm{moles}\;H}_{2}-{\mathrm{moles}\;\mathrm{CO}}_{2}}{{\mathrm{moles}\;\mathrm{CO}}_{2}+\mathrm{moles}\;\mathrm{CO}}$$

In ideal conditions, methanol production should require S = 2. The S number considers the continuous CO_2_ conversion with hydrogen into methanol via reverse WGS reaction. However, third parameter strictly depends on the adopted feedstock and this is the reason why, for example, if syngas is obtained from natural gas reforming, the S value ranges from 2.8 to 3. The second step to convert syngas into crude methanol is performed in the ranges 50–100 bar and 200–300 °C and the process mechanism is resumed below:

CO + 2H_2_ = CH_3_OH. (8)

Reaction (8) is subdivided in two consecutive steps:

CO + H_2_ = CH_2_O, (9)

CH_2_O + H_2_ = CH_3_OH. (10)

In addition to Reaction (8), methanol is also produced by Reaction (11):

CO_2_ + 3H_2_ = CH_3_OH + H_2_O. (11)

For the traditional gas phase process, the H_2_/CO ratio changes from 3:1 to 5:1 and the process requires a WGS stage to boost the hydrogen content. By contrast, for the liquid phase process, its superior heat management capabilities may handle the synthesis gas straight from the generator, with a ratio between 1:1 and 1:2, as generated by coal gasifiers [9]. Another issue to be considered is the thermodynamic equilibrium regulating the conversion of synthesis gas, which limits the process to low conversion and, consequently, imposes the recycling of a high amount of unconverted gas. Recycling and cooling duty are the main investment costs of this step process, although the continuous research in this field has allowed several solid catalysts to be developed, able to maximize methanol yield and selectivity, while lowering the formation of byproducts.

#### 2.1.1. Methanol Production through BASF Process

In the second decade of the 20th century, the hydrogenation of carbon monoxide giving methanol over iron-based catalysts at 500 °C and high pressure (100 bar) was pursued by Mittash et al. [26]. Successively, they performed methanol production using waste gases derived from ammonia synthesis, observing that this reaction produces various products in addition to methanol (Table 1), unfortunately thermodynamically more favored with respect to the latter [11,22]. Another key issue was the low methanol yield caused by pollutants present in the reactant gases, which were responsible for the deactivation of the Fe-based catalyst, the reaction between CO and Fe themselves, and the consequent penta-coordinate complexes formation [11].

**Figure 3:**
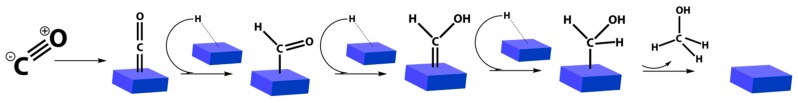
Scheme of the first reaction mechanism in four stages; the areas in blue color represent the catalyst surface.

Therefore, a part from the eighth group of the periodic system, many oxide- and metal-based catalysts were used for the hydrogenation reaction, carrying out this process at 250–300 bar and temperatures between 320 and 450 °C. At these operating conditions, the experimental results given by ZnO/Cr_2_O_3_ and ZnO/CuO catalysts were particularly interesting [9,22,26,27,28,29]. Based on the aforementioned solid catalysts, two ways were proposed for describing the reaction mechanism, both involving CO and H_2_ adsorption [30,31,32,33]. Figure 3 illustrates a scheme of the reaction mechanism taking place in four consecutive hydrogenation steps.

**Figure 4:**
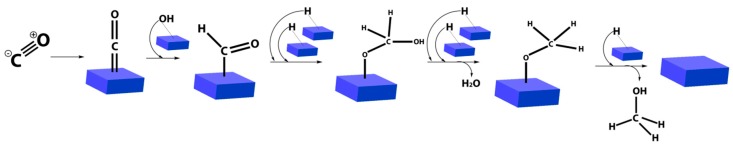
Scheme of the second reaction mechanism considering hydrogenation and dehydration reactions.

Figure 4 illustrates that, in the second proposed mechanism, both CO and a hydroxyl group are present on the catalyst surface, with the CO generating a formate intermediate; successively, hydrogenation and dehydration reactions are responsible for methanol production through a methoxide intermediate. These two mechanisms are completely different, firstly because they do not form the same intermediates and, secondly, because the latter is differently bonded to the catalyst surface. In the mechanism of Figure 3, they are bonded with C atoms, whereas in that of Figure 4 to O atoms.

**Figure 5:**
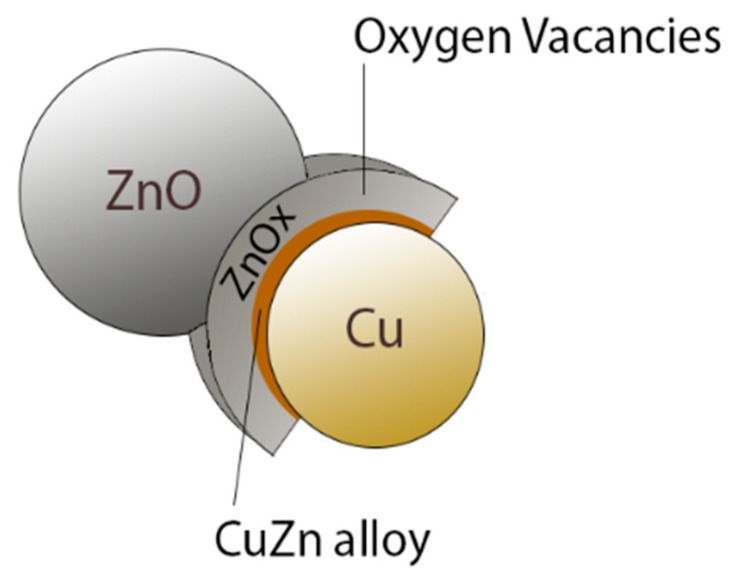
Schematic representation of oxygen vacancies in a Cu/ZnO catalyst.

In recent years, ZnO/Cu-based catalysts were the main subject of several scientific studies [27,28,34,35,36,37,38]. The mixture between Cu and ZnO creates small aggregates, in which ZnO is aggregated around Cu, forming a core–shell structure responsible for the selectivity of methanol production. This aggregate is formed partially by Cu, part of the shell comprises a Cu/Zn alloy, and the outermost part of the shell is represented by ZnOx (selective to the formation of methanol), where the vacations of oxygen are located, while another part of the aggregate consists of ZnO (Figure 5).

**Figure 6:**
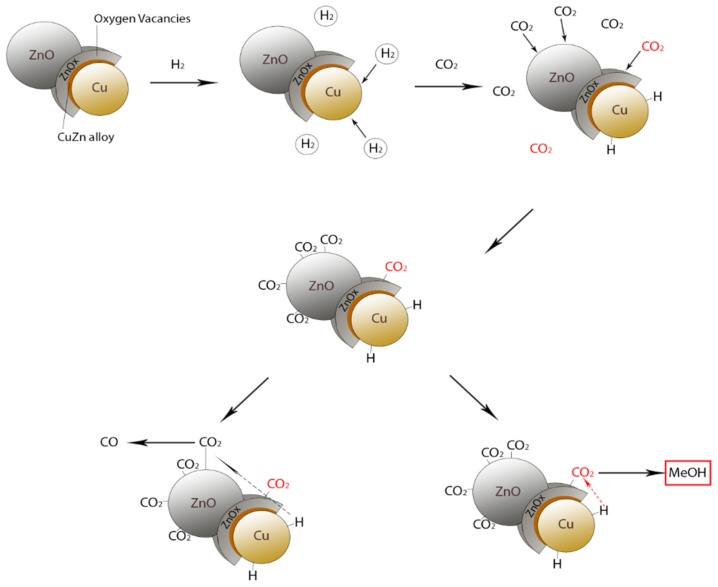
Schematic representation of the Cu/ZnO catalytic mechanism for methanol synthesis.

Tisseraud et al. [37] hypothesized that the morphostructural arrangement of the molecular aggregate plays a significant role in the catalyst’s selectivity towards methanol. In this structure, both H_2_ and CO_2_ are selectively chemisorbed on different surfaces of the catalyst. In fact, Cu does not adsorb CO_2_, but adsorbs hydrogen, albeit weakly, while CO_2_ is adsorbed on the surface of ZnO and ZnOx. As already reported in literature [27], H_2_ is weakly chemisorbed on metallic Cu due to its well-known characteristic to rapidly dissociate and recombine hydrogen. The weakly adsorbed H_2_ is then poured both on the ZnOx (leading to the formation of methanol) and ZnO part (leading to the formation of CO). A schematic representation of the Cu/ZnO catalytic mechanism is hence shown in Figure 6.

Tisseraud et al. [37] also reported a Cu-ZnOx/ZnO catalyst design; the ZnOx active phase should completely cover the Cu-sized particles, thus avoiding CO formation (unwanted product), making the catalyst highly selective toward methanol formation.

#### 2.1.2. Methanol Production through ICI PROCESS

**Figure 7:**
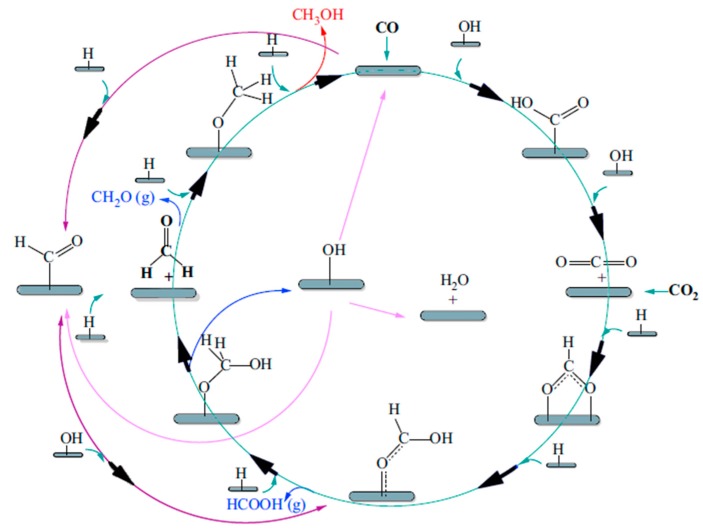
Scheme of a microkinetic model of a reaction mechanism for methanol synthesis over a Cu-based catalyst. With permission of reprint of Elsevier from Reference [2].

The hard experimental conditions imposed by the BASF process induced strong efforts to realize methanol synthesis at lower pressures. This was achieved by ICI Company (today, Johnson Matthey), which proposed a new methanol synthesis process in the pressure range from 35 to 54 bar and at temperatures from 200 to 300 °C. This goal was also due to the development of new Cu-based catalysts (Cu/ZnO/Al_2_O_3_) [28,29,30], but also to the development of new advanced separation/purification processes of synthesis gas, with the advantage of using sulfur–chlorine-free syngas [31,32,33]. However, although Cu/Zn-based catalysts proved to be very efficient in the methanol synthesis process, their wide utilization was negatively affected by their limited lifetime and low thermal stability [34]. These issues were overcome by adding alumina to the catalysts, responsible for the enhancement of catalytic stability, while depleting the Cu crystallites’ thermal formation [34,35,36]. Among several kinds of catalytic systems, Cu/ZnO/Al_2_O_3_-based catalysts seem to be the most promising ones. Liao et al. [39] reported significant morphology-dependent Cu–ZnO interactions, while Sun et al. [28] revealed their promotional effects on the catalytic performance of CO_2_ hydrogenation to methanol. A catalytic cycle is then proposed as a microkinetic model in Figure 7, which shows the scheme of a reaction mechanism where methanol may be generated by CO hydrogenation by means of HCOO, HCOOH, CH_2_COOH, CH_2_O, CH_3_O, or HCO, CH_2_O, CH_3_O intermediates [39].

It is worth of noting that, among various methanol production processes, differences in methanol yield (expressed in t/d) exist, depending on the feedstocks used; see Table 2.

As shown in this table, based on syngas as a primary feedstock the methanol yield varies from around 7.9 × 10^−2^ t/d with the BASF process to 2.4 × 10^3^ t/d with the Haldor Topsoe process, while the ICI process makes it possible to reach 2.5·× 10^3^ t/d using carbonaceous feedstocks.

### 2.2. Biomass and Char as Feedstocks for Methanol Synthesis

The use of biomass and char as feedstocks for methanol synthesis represents a new industrial way to solve the issues of energy demand, waste management, and pollutants emissions [40,41,42]. In the case of coal and biomass exploitation to produce methanol, the synthesis process is also similar to the one based on natural gas. In fact, it also foresees three stages, such as syngas production, crude methanol synthesis, and purification. At first, coal or biomass react in a gasifier to be converted into gaseous products, consisting of biogas (mainly CH_4_ and CO_2_), syngas (H_2_, CO_2_, and CO), hydrogen, and alkaline gases [43]. The gasification process represents a well-known thermochemical technique, useful for converting solid biomass into gaseous mixtures by means of air/oxygen, steam, and flue gases, and it is normally carried out at 800–1000 °C [33,44].

Otherwise, an alternative process to conventional gasification is represented by the torrefaction or “mild pyrolysis”, which allows the biomass properties’ optimization, making the combustion of biomass possible at temperatures ranging between 200 and 300 °C in anaerobic conditions. Indeed, at this operating temperature, water and other volatile compounds evaporate, leaving the lignocellulosic fraction under degradation with a consequent loss of the initial mass around 20% [31,45]. When methanol is synthesized from syngas via conventional gasification of biomass, the latter may be represented by whatever feedstock containing carbon, such as coal or solid wastes. However, conventional biomass gasification processes do not guarantee a syngas stream which is useful for methanol synthesis, due to the formation of tar and char via reduction of carbon oxides. Consequently, the requirements of synthesis gas which is useful for methanol production foresee a small portion of inert gases.

The catalysts used for producing methanol from syngas require a CO_2_:CO:H_2_ ratio = 5:28:63 [46], while the stage of syngas purification from substances which are environmentally harmful [47,48] is normally realized prior to the entrance of gases in the methanol production process.

### 2.3. CO_2_ Hydrogenation for Methanol Generation

The hydrogenation of CO_2_ to produce methanol (Equation (11)) may represent a viable green strategy for a sustainable development, oriented to produce added-value chemicals while reducing CO_2_ emissions in the atmosphere [49]. The utilization of CO_2_ as a feedstock includes several benefits because it is inexpensive, abundant in nature, nontoxic, noncorrosive, and nonflammable and, therefore, safe to handle. Furthermore, it may be easily stored and its transportation may be realized in liquid form under mild pressure. Methanol production from CO_2_ is hence advantageous not only for the exploitation of non-fossil fuel sources (unlike syngas), but also because it overcomes CO_2_ sequestration, which is a very costly process, also mitigating the GHG effect through an efficient recycling of CO_2_. Hence, CO_2_ could be separated and stored from human or industrial activities as well as by absorption from air, then converted renewably into methanol. The conversion of CO_2_ into methanol via hydrogenation reaction (Equation (11)) requires a considerable energy supply; consequently, an adequate catalytic system is needed. Considering the actual importance and interest on the CO_2_ hydrogenation, a recent progress about its challenges and opportunities may be found in Li et al. [50].

This process is conventionally carried out from 35 to 55 bar and 200 °C, while the highest active and selective catalysts are based on Cu/ZnO/Al_2_O_3_. The whole reaction pathway for the methanol production via hydrogenation reaction is reported below:

CO + 2H_2_ = CH_3_OH, (12)

CO_2_ + H_2_ = CO + H_2_O, (13)

CO_2_ + 3H_2_ = CH_3_OH + H_2_O. (14)

From a theoretical point of view, to enhance methanol production in conventional reactors, two main routes (apart from the catalyst improvements) can be also pursued:

(a) Recycling of the unconverted synthesis gas after products separation by condensation;
(b) in situ reaction products removal.

However, the catalytic hydrogenation of CO_2_ using H_2_ produced with renewable energy is considered a potential path forward for the sustainable production of methanol, but also of lower olefins, higher hydrocarbons, formic acid, and higher alcohols. Furthermore, CO_2_ hydrogenation represents a promising way to convert it into fuels, among other CO_2_ hydrogenation paths, needing to evaluate two challenges along with it: (a) Sustainable hydrogen source and (b) dispersed product distribution [51].

Much effort was devoted to solving the former challenge and many scientists have already made great progress in water electrolysis to produce H_2_ using electricity (Equation (14)) generated with solar or wind or other renewable energy, and water splitting using photocatalytic, photoelectrochemical, or other photochemical processes [52].

2H_2_O → 2H_2_ + O_2_ (15)

Nevertheless, at the moment, H_2_ is produced expensively and not renewably in conventional systems, representing a strong issue for the development of this process at a larger scale [37].

## 3. Methanol Utilization

In 2010, global methanol demand achieved 49 million tons and, according to the IHS Markit World Analysis—Methanol 2017, by 2021 the demand will overcome 95 million tons, with China playing the role of leader in this market, with around 54% of world capacity and 46% of global production. It is estimated that, by 2021, nearly one in five tons of global methanol production will be useful for the methanol-to-olefins process and to satisfy the Chinese chemical demand. In 2000, China represented 12% of global methanol demand, while North America and Western Europe 33% and 22%, respectively. In the previsions of IHS Markit, Northeast Asia (dominated by China) will account for around 70% of global methanol demand, followed by North America with 9% and Western Europe with 8% [53].

### 3.1. Methanol Transformation into Dimethylether

Among other chemicals, methanol utilization for di–methyl–ether (DME) production has received growing interest in the last decade. Indeed, methanol plays an important role as a C1 building block in the petrochemical industry and a consistent fraction of its production is consumed in the manufacturing of DME [54,55], as well as an alternative fuel for automotive applications. DME has an octane number and ignition temperature similar to those of diesel fuel, showing interesting characteristics, such as lower NO_X_ emission and less smoke and engine noise than those of conventional diesel engines; furthermore, it can be handled easily [56].

The current transformation of methanol into DME is performed by a double stage (indirect) synthesis, which is followed by dehydration reaction as represented by Equation (15).

2CH_3_OH → CH_3_OCH_3_ + H_2_O (16)

A further process involves the direct synthesis of DME from syngas, which proceeds with a syngas having CO:H_2_ molar ratio equal to 1:1, as described in the reaction reported below [57]:

3CO + 3H_2_ → CH_3_OCH_3_ + CO_2_. (17)

### 3.2. Methanol in Fuel Cells Applications: DMFCs

**Figure 8:**
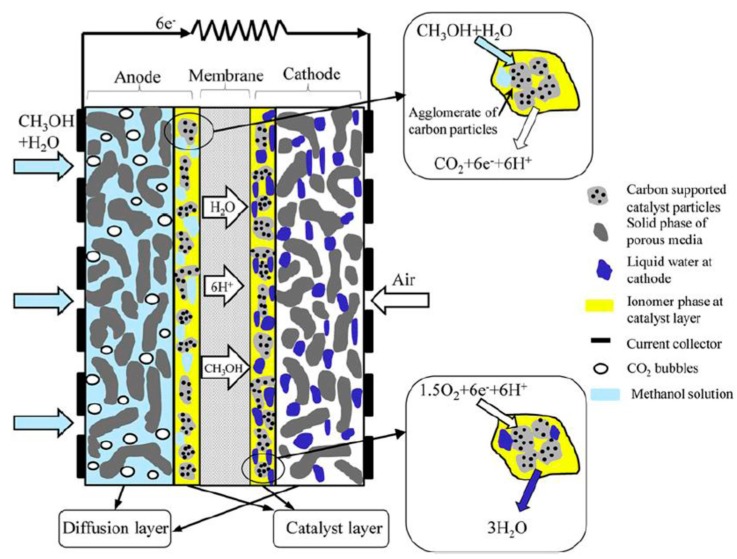
Schematic view of a direct methanol fuel cell (DMFC). With permission of reprint of Elsevier from Reference [59].

A direct methanol fuel cell (DMFC) is classified as a low temperature polymer electrolyte membrane fuel cell supplied by liquid or vapor methanol [58]. Figure 8 shows the schematic diagram of a DMFC, which consists of at least five main porous layers:

(1) Anode gas diffusion layer (AGDL),
(2) anode catalyst layer (ACL),
(3) polymer electrolyte membrane,
(4) cathode catalyst layer,
(5) cathode gas diffusion layer.

The methanol fed diffuses through the AGDL towards ACL. Methanol is oxidized to CO_2_ (Equation (15)) at anode side, allowing six H^+^ to pass through the membrane and six e^−^ across the external circuit, generating electricity. At the cathode side, oxygen from air is reduced to water through the diffusion layer and catalyst layer. The membrane acting as an electrolyte is coated with a catalyst, enhancing the oxidation of methanol at the anode side and the reduction of oxygen at the cathode side.

Anode: CH_3_OH + H_2_O → CO_2_ + 6H^+^ + 6e^−^ (18)

Cathode: 1.5O_2_ + 6H^+^ + 6e^−^ → 3H_2_O (19)

Overall: CH_3_OH + H_2_O + 1.5O_2_ → CO_2_ + 3H_2_O (20)

DMFCs may be subdivided, depending on the way of fuel and oxidant supply as (a) active, (b) passive and (c) semi-passive.

Conventional active DMFCs operate by means of auxiliary devices, such as fuel feed pump, oxidant supplier, CO_2_ separator, and fuel cell stacks.

By contrast, passive (air-breathing) DMFCs do not operate by auxiliary devices; hence, they need natural mechanisms, such as convection, capillary, gravity, concentration gradient, or osmosis, to deliver fuel and oxidant. Air is supplied by a breathing mechanism to cathode, while methanol is supplied to anode from a reservoir with a concentration gradient between anode and reservoir [60].

Semi-passive DMFCs represents a particular class able to combine active and passive typologies, attracting a growing attention for the development of compact portable devices with power below 10 W [59]. As an example, a number of big companies, such as Motorola, Toshiba, Samsung, and NEC, were involved in the research, development, and application of passive DMFCs [61]. Among others, Toshiba applied a DMFC as mobile devices, requiring manually filling methanol solution from a cartridge and obtaining oxygen directly from ambient air (palm sized Dynario^TM^) [62].

Nevertheless, DMFC technology also shows some drawbacks when it comes to its wide diffusion. In particular, even though Nafion utilization has been largely investigated as a polymeric membrane, methanol crossover represents its main negative aspect, being responsible for loss fuel and lower overall cell voltage, limiting the application of DMFCs [63]. Therefore, the research in this field is devoted to analyzing valid options to Nafion membranes to reduce the effects of methanol crossover and to allow a wider utilization and commercialization of DMFCs [64].

## 4. Methanol Exploitation for Hydrogen Generation

As affirmed by the International Energy Agency, today the world economy is still depending on the exploitation of fossil fuels, such as oil, coal, natural gas, etc. [65]. Consequently, the increment of the environmental pollution due to CO_2_ emissions and other dangerous gases originating from burning fossil fuels is driving both industry and academia to research new and green technologies, and renewable feedstocks exploitation as well. Hydrogen is currently seen as a clean energy source, playing a relevant role in refining, chemical, and electronic industry [66]. However, hydrogen presents some drawbacks in terms of the difficulties in its storing and transportation, negatively affecting its wider utilization. Consequently, hydrogen generation from an easily transported liquid source may represent a valid option. Methanol is considered an excellent candidate as a hydrogen carrier, showing low toxicity and easy handling. In the following, different industrial processes involving methanol for hydrogen generation are reported and discussed.

### 4.1. Methanol Decomposition Reaction

According to Equation (21), a methanol decomposition (MD) reaction is an endothermic process able to generate H_2_ and CO:

CH_3_OH = CO + 2H_2_ (21)

This process is convenient for recovering industrial heat waste at around 200 °C [67]. It is particularly important to mention that active catalysts based on palladium and prepared by co-precipitation are able, even at temperatures below 200 °C, to catalyze this reaction.

### 4.2. Methanol–Water Solution Electrolysis

**Figure 9:**
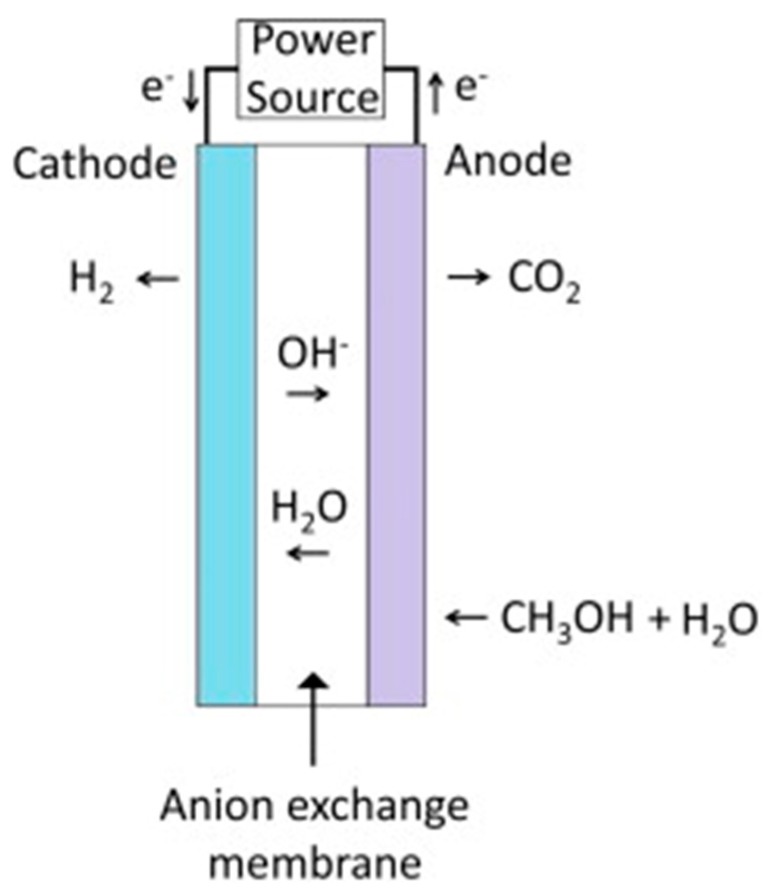
Schematic view of methanol–water solution electrolysis. With permission of reprint of Elsevier from Reference [70].

Methanol–water solution electrolysis is another process to generate hydrogen using an electrolytic cell. Commonly, the water electrolysis process represents the best way to produce high grade hydrogen quickly [68]. As a consequence, also using a methanol–water solution, hydrogen may be easily produced with a purity varying between 95% and 97%, even though the theoretical voltage of the system is much lower (0.03 V) than in water electrolysis (1.23 V) [69]. During methanol–water solution electrolysis, hydrogen is generated by applying a direct current to the electrolytic cell, as shown in Figure 9. At the anode side, methanol and water react, generating CO_2_, protons, and electrons. Protons migrate through the polymeric membrane from the anode to the cathode of the electrolytic cell, while electrons migrate to the cathode through the external circuit containing the direct current power supply. Hydrogen generation from methanol–water solution electrolysis is useful for portable power applications, since the start up and shut down are possible very quickly and at relatively low temperature [68].

### 4.3. Methanol Steam Reforming Reaction for Hydrogen Generation

Methanol steam reforming (MSR) has been largely investigated and, currently, the most active catalysts for this process are based on Cu, which are cheap and normally operated in the temperature range from 240 to 260 °C [71].

CH_3_OH + H_2_O = CO_2_ +3H_2_ (22)

Nevertheless, Cu-based catalysts suffer low stability and a pyrophoric nature and, furthermore, are responsible for a significant production of CO. Catalyst deactivation is normally due to sintering, coke deposition, catalyst poisoning (chloride, sulphur), and change in oxidation state. The prevention of coke formation can be done using an excess of water in the feed and, generally, the best results are obtained for molar water/methanol ratio of 1.5:1 [72,73].

Consequently, the research on MSR catalysts which are more active, stable, and responsible for a lower CO production has been greatly pursued. In particular, catalysts able to be operated at around 180 °C should represent a desired goal (indeed, at this temperature, higher stability and low CO production are expected) and, from a thermodynamic point of view, they should show almost complete conversion. In the viewpoint of fuel cells and fuel processors integration, being MSR an endothermic reaction, the reformer reactor could be synergistically coupled with a high temperature proton exchange membrane fuel cell (HT-PEMFC), which works exothermally in the range from 160 to 180 °C.

Apart from Cu-based ones, other catalysts were used for the MSR reaction, in particular those based on palladium, which—compared to nickel, platinum, ruthenium supported on ZnO—showed lower CO production and higher conversion [74]. Additionally, the utilization of bimetallic catalysts seems to be an interesting choice to enhance both activity and selectivity. Among others, the best activity reported was achieved using Pd/Zn and Pd/Ga bimetallic catalysts and the best selectivity with Pd/Cd [75].

Regarding the typology of the MSR reactor design, it is worth noting that it could play a direct role in the reaction performance. Therefore, the reactor design has as a target to be as cheap as possible and to maximize both conversion and selectivity, also taking into account that its process performance is influenced by the flow pattern, velocity profile, pressure drop, and heat transfer [76]. Most of the reactor designs deal with rectilinear channels, pinhole, coil-based, and radial, even though conventional reactors (CRs) are currently tubular due to the complexity and higher costs of realizing the other configurations. Nevertheless, recently, the area of micro fuel processors made it possible and easier to realize further reactor designs and, namely, well-structured flat microreactors, showing benefits over the conventional ones, such as higher surface-to-volume ratio, smaller mean distance of the specific fluid volume to the reactor walls, better heat and matter transfer properties, and flow patterns that fit with the reaction needs. A microreactor is defined as a device that contains micro structured features, with a sub-millimeter dimension, in which chemical reactions are performed in a continuous manner [77]. Compared to the CRs, microreactors present several benefits, such as enhanced heat and matter transfer properties, reduced mean distance of the specific fluid volume to the reactor walls, higher surface-to-volume ratio, etc.

As a further subdivision, it is possible to consider microreactors ranging from 0.1 to 10 cm^2^ in area and mini-reactors between 10 and 200 cm^2^ [78]. The latter, however, seem to be more adequate for packed bed applications, better combining the requests of the typical fuel cells size.

### 4.4. Process Intensification Strategy Applied to MSR Reactors

**Figure 10:**
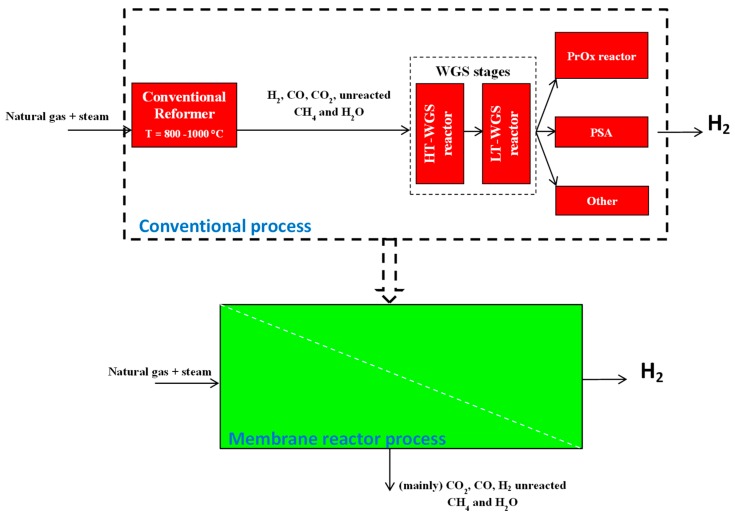
High grade H_2_ generation from natural gas steam reforming: Conventional vs. membrane reactor process.

The MSR reaction originates from a stream containing, in addition to hydrogen, other byproducts, such as CO, CO_2_, and small amounts of reactants. Therefore, in the viewpoint of combining a fuel processor and PEM fuel cells, hydrogen needs high purity prior to entering in the PEMFC. As for the industrial natural gas steam reforming to produce hydrogen, the reformed stream coming from an MSR reactor should need further hydrogen purification/separation processes, namely water gas shift reaction performed in two reactors operating in series at high and low temperature, partial oxidation reactor to convert CO into CO_2_, and pressure swing adsorption for separating CO_2_ from H_2_. As is well known, the aforementioned stages of hydrogen purification impose heavy costs and negatively affect the overall process efficiency. Thus, according to the principles of Process Intensification Strategy, today it is well recognized that membrane reactor (MR) technology plays an important role as a valid option to the CRs, because it is able to combine the reforming reaction for generating hydrogen and its purification in a single stage without needing further hydrogen separation stages (Figure 10) [79,80].

The concept of MR technology was first introduced in the 1950s, although the utilization of new inorganic materials and the development of high-temperature membrane processes have received much attention in the last three decades. In the field of MR utilization for hydrogen generation, a general subdivision of MR applications can be summarized as reported below:

(a) Inorganic membrane reactors [79,81];
(b) self-supported and supported Pd-based membrane reactors [80,82];
(c) Zeolite membrane reactors [83];
(d) biomembrane reactors [84];
(e) photocatalytic membrane reactors [85].

However, most of the open literature dedicated to hydrogen generation via MR technology is devoted to the investigation of inorganic membranes application [82,86,87,88,89]. Currently, the operating modality of membranes housed in a MR can be subdivided as reported below:

- Extractor modality: The membrane selectively removes hydrogen from the reaction mixture for permeation.
- Distributor modality: The membrane allows the controlled addition of hydrogen to the reaction mixture.
- Contactor modality: The membrane emphasizes the contact within reactants and catalyst.

In the next sub-paragraphs, special attention is dedicated to the characteristics and hydrogen separation/purification performance of palladium-based membranes and their utilization in MRs for producing high grade hydrogen, the latter combining in an integrated/intensified whole process the chemical reaction for transforming methanol via reforming reaction into hydrogen and the hydrogen separation/purification stage in an unique system.

### 4.5. Applications of Pd-Based Membranes in Membrane Reactors

**Figure 11:**
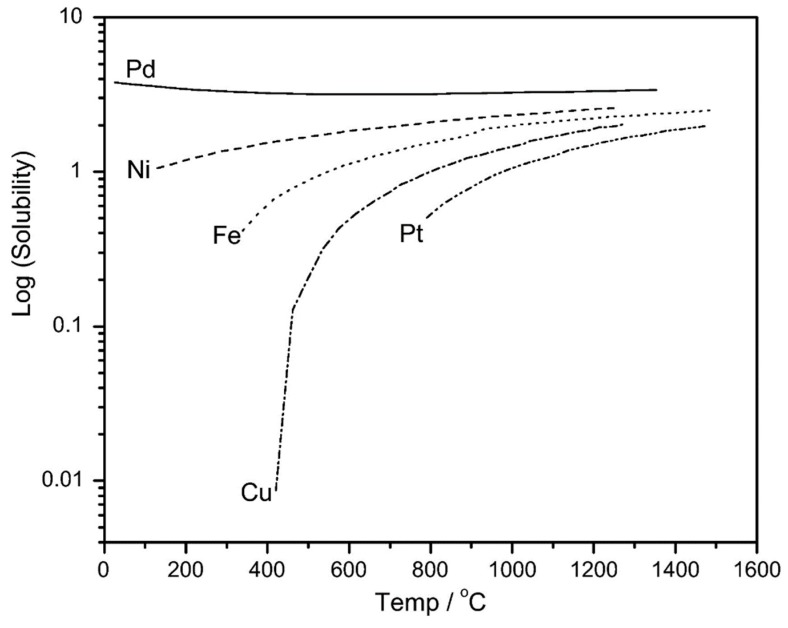
Hydrogen solubility vs. temperature in different metallic materials. With permission of reprint of Elsevier from Reference [89].

Metallic-based membranes are presently usually applied in gas separation and in MR applications [79,82]. In particular, palladium and its alloys represent the dominant materials because they possess high H_2_ solubility, compared to other metallic materials (Figure 11), which makes them useful for preparing H_2_ perm-selective membranes [89].

The hydrogen transport mechanism through dense Pd and/or Pd-alloy is described by the solution–diffusion, taking place in six steps as reported here below:

1. Hydrogen molecules adsorption from the membrane;
2. dissociation of hydrogen molecules on the membrane surface;
3. reversible dissociative chemisorption of atomic hydrogen;
4. reversible dissolution of atomic hydrogen in the metal lattice of the membrane;
5. diffusion into the metal of atomic hydrogen proceeds from the higher hydrogen pressure to the lower hydrogen membrane side;
6. desorption of recombined atomic hydrogen into molecular form.

Hence, hydrogen permeation through a Pd-based membrane may be described by Equation (23):

(23) $$J_{H_{2}}=\frac{P_{H2}\left(p_{hps}^{n}-p_{lps}^{n}\right)}{\delta }$$

where *J_H_*_2_ is the hydrogen permeating flux, *P_H_*_2_ the hydrogen permeability, *δ* the thickness of the palladium/palladium alloy layer, *p_hps_* and *p_lps_* are the hydrogen partial pressures on the high pressure (feed) and low pressure (permeate) sides, respectively, while “*n*” is the pressure exponent. The *n*-value can vary between 0.5 and 1, depending on the rate-determining step among the aforementioned (a)–(g) stages. If the rate-controlling step is represented by bulk diffusion through the palladium layer (c), then *n*-value is equal to 0.5 and Sieverts law is followed (usually valid for thick palladium films, >5 μm) (Equation (24)) and, consequently, the membrane shows full hydrogen perm-selectivity.

(24) $$J_{H_{2}}=\frac{P_{H2}\left(p_{hps}^{0.5}-p_{lps}^{0.5}\right)}{\delta }$$

Otherwise, in the case of mass transport to or from the surface (a,g) or dissociative adsorption (b) or associative desorption (e) representing the rate determining stage, *n*-value becomes 1, indicating that the processes depend linearly on the concentration of molecular hydrogen. Commonly, *n* = 1 suggests that the permeation through the palladium is very fast (particularly for palladium layer <5 μm).

For thick palladium films, deviation from Sieverts law (*n*-value > 0.5) can be induced by high hydrogen pressure, decrease in the surface reaction rate after absorption of contaminants, concentration polarization and defects due to pinhole formation. Consequently, hydrogen passes through the Pd-based layer not only for the solution–diffusion mechanism, but also via Knudsen or viscous flow mechanisms (Equation (25)) [89]:

(25) $$J_{H_{2}}^{Total}=\frac{1}{\frac{1}{J_{H_{2}}^{SD}}+\frac{1}{J_{H_{2}}^{K}}+\frac{1}{J_{H_{2}}^{HP}}}$$

with $J_{H_{2}}^{Total}$ representing the total hydrogen permeating through the membrane, $J_{H_{2}}^{SD}$ the hydrogen permeating flux via solution/diffusion mechanism, $J_{H_{2}}^{K}$ the hydrogen permeating flux via Knudsen mechanism, and $J_{H_{2}}^{HP}$ the hydrogen permeating flux via viscous flow/Hagen–Pouiselle mechanism.

**Figure 12:**
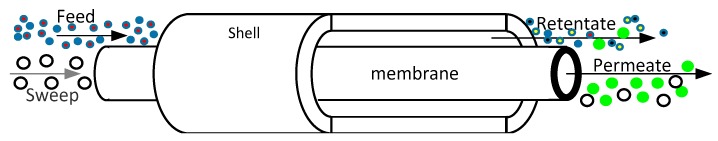
General scheme of a membrane reactor housing a tubular H_2_ perm-selective membrane.

Therefore, the combination of a hydrogen perm-selective MR and a reforming process (such as MSR reaction) may produce various synergistic advantages. Indeed, the continuous H_2_ removal from the reaction side shifts the reaction toward further product formation, allowing it to reach higher conversion (the so-called “shift effect”), while recovering a high grade hydrogen stream in the permeate side, as shown in Figure 12.

### 4.6. Methanol Steam Reforming Reaction in Membrane Reactors for Hydrogen Generation

In the last two decades, the interest in MSR reaction combined to MRs was reflected in the consistent number of scientific studies present in the open literature, particularly because this combination may offer various benefits for producing hydrogen with respect to the conventional low-pressure systems. Nevertheless, the cost of palladium represents a crucial issue for proposing Pd-based membrane systems as a mature technology, ready for entering in the market. Consequently, taking into account the intrinsic high cost of palladium, the scientific community was oriented to adopt membrane solutions with reduced Pd-content. Table 3 shows some of the most representative results in terms of conversion, hydrogen recovery and purity, operating conditions, etc. related to MSR reaction performed in MRs [90,91,92,93,94,95,96,97,98,99,100,101,102,103,104,105,106]. In particular, this table reports the recent advances in composite Pd-based membranes (a dense, thin layer of Pd and/or its alloy deposited on a porous substrate), even considering not Pd-based membranes in order to highlight benefits and drawbacks between the different solutions.

In detail, Lytkina et al. [90] prepared two dense unsupported Pd–Ag and Pd–Ru membranes via magnetron sputtering, to be housed in a MR for carrying out an MSR reaction at 300 °C and 1:1 feed molar ratio. Reduced methanol conversions were achieved, although reaching a hydrogen recovery of 38% and 18%, respectively, with a purity of around 100%. Saidi [91] performed MSR in a self-supported Pd–Ag (6 μm thick) MR at 300 °C and 2 bar using a Cu/ZnO/Al_2_O_3_ catalyst, reaching 98% of conversion, while recovering more than 60% of hydrogen.

Liguori et al. [92] used a supported Pd/PSS MR packed with a CuO/ZnO/Al_2_O_3_ catalyst; the composite membrane was prepared by electroless plating deposition (ELP), showing an average metallic layer of around 7 μm. At 330 °C and 2.5 bar, they reached 85% of methanol conversion and 40% of hydrogen recovery, with a purity of around 100%. Another kind of composite membrane (Pd–Ag/TiO_2_-Al_2_O_3_) was used by Basile et al. [93] for performing an MSR reaction. Nevertheless, the presence of defects in the separative layer was responsible for poor performance, although operating at a relatively higher temperature (550 °C): 65 of methanol conversion with a recovered hydrogen stream showing a purity of 72%. Lin et al. [94] used a supported Pd/PSS membrane, prepared by ELP deposition, which showed a metallic layer of 20 μm. In this study, an MSR reaction was performed at 350 °C, 6 bar and 1.2:1 feed molar ratio over a Cu-based catalyst, reaching methanol conversion of 95%, 97% of hydrogen recovered in the permeate stream with a purity of 100%. In another work, Lin’s group [97] used a MR allocating a Pd/PSS membrane with the Pd-layer of 20–25 μm. At 350 °C, a methanol conversion higher than 99% and pure hydrogen recovered in the permeate stream were reached.

Israni and Harold [95] carried out an MSR in a MR housing a thin Pd–Ag/Al_2_O_3_ membrane, having a separative layer of around 4 μm, prepared by ELP deposition. At 250 °C and stoichiometric feed molar ratio, they got complete conversion with a hydrogen recovery ranging from 45% at 2.5 bar to 95% at 10 bar, with a hydrogen purity ~100%. Poor results were reached by Rei et al. [96], who used a 20–25 μm thick Pd–Ag/PSS MR. At 240 °C and 10 bar; less than 40% of methanol conversion was obtained, with a poor hydrogen recovery (18%). This probably occurred because the supported membrane showed defects in the separative layer, which negatively affected its perm-selectivity characteristics, determining a low shift effect on MSR reaction, with a consequent low performance. Iulianelli et al. [98] performed an MSR reaction in a self-supported Pd–Ag MR at 300 °C, 3 bar, and feed molar ratio = 3:1 over a CuO/Al_2_O_3_/ZnOMgO catalyst. The membrane showed a thickness of 50 μm and was prepared by the cold-rolling technique, allowing a hydrogen recovery of 80%, having a purity of 100%. Also Itoh et al. [99] housed in a MR a self-supported membrane based on Pd–Ru–In (200 μm thick), reaching, at 200 °C and 7 bar, a methanol conversion of 90% and a hydrogen recovery of 24%. The latter result was probably due to the high thickness of the membrane, which was responsible for low hydrogen permeability and, consequently, determined a reduced removal of hydrogen from the reaction to the permeate side. Nevertheless, the recovered hydrogen showed 100% of purity.

Wieland et al. [100] housed different self-supported Pd-alloyed membranes (Pd–Ag, Pd–Cu. and Pd–V–Pd) in MRs for performing an MSR reaction. Among them, the best performance was reached using a Pd–Cu membrane (25 μm thick), which proved the most stable; at 300 °C and 10 bar, they obtained a methanol conversion which was higher than 90%, recovering around 40% of pure hydrogen.

Table 3 does not report only interesting data about the application of Pd-based membranes in MRs, but also alternative and cheaper solutions with respect to palladium, namely carbon and silica membranes. In particular, Sà et al. [101] realized a carbon membrane via the pyrolysis technique, starting from dense cellulose cupra–amonia hollow fibres. It showed relatively low H_2_/N_2_ perm-selectivity, but housed in a MR for carrying out an MSR reaction at 200 °C and 1 bar made it possible to reach a conversion of >90% and a hydrogen recovery of >80%. Briceño et al. [102] prepared a supported carbon membrane on a porous ceramic substrate, constituted of TiO_2_ coated with ZrO_2_. Hence, different polymeric solutions were deposited as carbon precursors and successively pyrolyzed. The supported carbon membrane was housed in an MR for an MSR reaction and methanol conversion of >50% was obtained at 250 °C and 2 bar, while recovering a hydrogen stream with a purity of ~80% [103]. Zhang et al. [104] allocated in an MR a carbon membrane in a tubular shape with 6-mm of internal diameter and wall thickness of ~20–30 μm, sealed inside a stainless steel tube. During MSR at 250 °C and 2 bar, methanol conversion was around 100%, with a recovery of hydrogen showing 97% of purity.

Lee et al. [105,106] developed two supported silica-based membranes (SiO_2_/γ–Al_2_O_3_/Pt–SiO_2_/PSS and SiO_2_/γ–Al_2_O_3_) membranes for carrying out an MSR reaction in an MR. The first membrane (SiO_2_/γ–Al_2_O_3_/Pt-SiO_2_/PSS) was housed in am MR module packed with a Cu–Zn-based catalyst at 230 °C. Complete methanol conversion was reached, although the recovery of hydrogen was very poor (~10%). By contrast, the utilization of the second membrane (SiO_2_/γ–Al_2_O_3_) operated in MR at 260 °C and feed molar ration 3:1 showed a lower conversion (~40%) and hydrogen recovery (5%), even though the purity of the recovered hydrogen was quite high (98%).

### 4.7. Photocatalytic Membrane Reactors: Methanol Production from CO_2_ Reduction

Today, photocatalytic membrane reactors (PMRs) are seen as an interesting option for conventional processes in the field of water and air purification and in organic syntheses as well. MR technology shows certain benefits, such as low energy and chemicals consumption, automatic control, and steady/easy operation, making it an ideal subject in several separation processes. Taking these advantages into account, hybrid processes combining photocatalysis and membrane operations are able to achieve a synergistic effect, minimizing environmental and economic concerns. PMRs are then considered a green technology, because they guarantee the safety of the photocatalyst used and operate at mild conditions and in continuous configuration, in which catalyst recovery, reaction, and products separation take place simultaneously, with consequent time- and cost-saving [107,108]. Furthermore, the possibility to combine the photocatalytic reactions with solar light utilization proved particularly attractive, making this process really interesting for future industrial applications.

In this scientific field, CO_2_ transformation into organic molecules like methanol has proven to be very attracting, because it is potentially useful for depleting CO_2_ emissions from industrial streams, obtaining added-value products, and constituting a great goal for the scientific community. In this regard, CO_2_ reduction by using TiO_2_ as a photocatalyst both in liquid and in gas phase was the main subject of various scientific studies [109,110,111]. Among them, Sellaro et al. [109] performed CO_2_ reduction into methanol in a PMR operated at mild conditions, in which TiO_2_ was immobilized in Nafion membranes. The reaction was performed under UV light in liquid phase using H_2_O as a reducing agent by housing the membranes in a flat sheet membrane module equipped with a quartz window to allow the irradiation. The proposed membrane configuration associated to the continuous flow modality made the removal of methanol from the PMR possible, avoiding its overoxidation.

Pathak et al. [110] allocated a porous optically transparent ionomer Nafion membrane embedded with TiO_2_ nanoparticles in a PMR to carry out the photocatalytic reduction of supercritical CO_2_. They found that, among methanol, ethanol, and formaldehyde, their composition was determined by the flow rate and by the weight of TiO_2_ used.

More recently, Pomilla et al. [111] proposed CO_2_ reduction into fuels in a continuous PMR housing a Nafion membrane with embedded exfoliated C_3_N_4_. The PMR converted CO_2_ in various products, such as methanol, ethanol, acetone, etc., with compositions strongly dependent on H_2_O/CO_2_ feed molar ratio and residence time.

However, the knowledge about the fundamentals of photocatalysis represents a crucial aspect to better understanding all the parameters able to affect the process under investigation. In the near future, the development of new photocatalysts coupled with PMR utilization will constitute a critical task, keeping in mind clearly that a sustainable process may be pursued when a PMR is combined with the sun as a cheap and clean source of light.

## 5. Recent European Projects Involving Methanol Production, Utilization, and Transformation

The European scientific community as well as the European Commission have concretely contributed in recent years to the development of a methanol platform, in which the main subject of the various European Projects is methanol as a source and/or intermediate for its transformation in other added-value products. These European actions have been realized with the financial support of various scientific projects, briefly illustrated in this paragraph.

“MefCO_2_—Methanol fuel from CO_2_” has been funded by the European Commission within Horizon 2020 (2014–2020) projects and is coordinated by I-Deals/Every Group (Spain). It aims to produce green methanol as an energy vector from captured CO_2_ and hydrogen produced using renewable energy surplus. The technology is being designed in a modular intermediate scale, with the aim of being able to adapt it to varying plant sizes and gas composition.

“WOODSPIRIT” was a big demonstration project developed by the company BioMCN (The Netherlands) for the production of biomethanol, for use as a transportation fuel additive. The consortium got 199 million euros under the first call for proposals of the NER300 funding EU program for innovative low-carbon technologies, demonstrating the production of biomethanol in a large commercial scale (413,000 t/y) using biomass torrefaction and entrained flow gasification as the new core technologies. The aim of this project was to utilize the biomethanol as a petrol additive for partial replacement of mineral fuel.

“BEINGENERGY—Integrated low temperature methanol steam reforming and high temperature polymer electrolyte membrane fuel cell” has been a FP7/JTI-CP-FCH—Joint Technology Initiatives—Collaborative Project (FCH), realized in the period between 2012 and 2016 and coordinated by University Porto (Portugal). This project was aimed at proposing a power supply comprising a methanol steam reformer and high temperature polymer electrolyte membrane fuel cell (HT-PEMFC) operating at the same temperature. Additionally, it proposed the application of the Pd-based MR technology for single-stage hydrogen production and of an ionic-liquid-supported polymer membrane selective for CO_2_ removal from reformate streams operating at up to 200 °C.

In the same field of application, The European FP7 project “LIQUID POWER” was developed in the period from 2011–2016 and coordinated by Dantherm Power (Denmark), with the aim of developing a new generation of fuel cell systems for backup power markets and for material handling vehicles, as well as for developing new innovative hydrogen supply methods for onsite methanol reforming.

“CEOPS—CO_2_ loop for Energy storage and conversion to Organic chemistry Processes through advanced catalytic Systems” has been an NMP–FP7 European project devoted to a sustainable approach for the production of methanol from CO_2_, acting as a precursor for fine chemicals products. It was realized between 2013 and 2016 and was coordinated by the CEA Institute (France). The concept of the project was concentrated on the development of two chemical pathways based on: (1) CO_2_ to methane conversion, realized with advanced catalysts to promote the efficiency of the electro–catalytic process at the point of CO_2_ emission (cement works); (2) direct conversion of methane to methanol, realized with advanced catalysts to promote the efficiency of the direct pathway instead of using the current pathway consisting of a steam reforming of CH_4_, which represents 60–70% of cost of production of current methanol, followed by the CO hydrogenation reaction.

“NEMESIS2+—New Method for Superior Integrated Hydrogen Generation System 2+” has been an FCH–JU European Project developed from 2012 to 2015 and coordinated by German Aerospace Center—DLR e.V. (Germany). The principal objective of this project was the development of a small-scale hydrogen generator capable of producing 50 m^3^·h^−1^ hydrogen (purity: 5.0) from biodiesel and diesel. In detail, methanol was used as a reactant to convert triglyceride into Fatty Acid Methyl Ester, which currently represents biodiesel.

“SUPER METHANOL—Reforming of Crude Glycerine in Supercritical Water to Produce Methanol for Re-Use in Biodiesel Plants” was a CP–FP small- or medium-scale focused research project, developed between 2008 and 2011 and led by B.T.G. Biomass Technology Group BV (The Netherlands). The objective was to produce methanol from crude glycerine, reusing it in the biodiesel plant while improving the energy balance, carbon performance, sustainability, and overall economics of biodiesel production. In practice, the consortium was able to perform the reform of glycerine in supercritical water, and to produce a synthesis gas suitable for direct once-through methanol synthesis.

“METAPU—Validation of a Renewable Methanol-based Auxiliary Power System for Commercial Vessels” was an FP6–SUSTDEV-3—Global Change and Ecosystems EU project coordinated by Wärtsilä Corporation (Finland). This project investigated, in the period from 2006–2010, the use of methanol and solid oxide fuel cell technology for shipping. In particular, it allowed to successfully propose methanol utilization for fuel onboard ships, whereas international regulations permit only the carriage of methanol as cargo.

## 6. Conclusions and Future Trends

A comprehensive review on methanol production and application was reported in this work, paying particular attention to the exploitation of methanol through MR technology for hydrogen generation. In this work, it was clearly represented how methanol plays a key role as one of the most significative and versatile molecules, with interesting potentials as easily transportable fuel and in the chemical industry (as a solvent and as a C1 building block for producing intermediates and synthetic hydrocarbons) or as a convenient energy carrier for hydrogen generation. Indeed, methanol seems to be the most significant product of the future, both for green chemistry development and as a hydrogen carrier. In particular, the review highlighted the most important methanol production cycles coming from biomass and/or CO_2_, the latter case being particularly important in the viewpoint of the minimization of GHGs production and as a solution to global warming.

The second part of the review was related to the hydrogen generation via reforming reactions involving methanol as well as to the role of methanol in DMFC applications. Furthermore, the principles of the Process Intensification Strategy and the consequent application of MR technology were also discussed. Last but not least, a panoramic view on the most significative European Project developed in the last ten years about methanol was also added.

## Figures and Tables

**Table 1:** Reactions involved in methanol synthesis.

Long-chain alcohols	*n*CO + 2*n*H_2_ = C*_n_*H_2*n*+1_OH + (*n* − 1)H_2_O
Aldehydes Ketons	RCH_2_CH_2_OH = RCH_2_CHO + H_2_
	2RCH_2_CHO = RCH_2_COCHRCH_3_ + O_ads_
Hydrocarbons	CO + 3H_2_ = CH_4_ + H_2_O
	CO_2_ + 4H_2_ = CH_4_ + 2H_2_O
	*n*CO + (2*n* − 1)H_2_ = C*_n_*H_2*n*+2_ + *n*H_2_O
Dimethyl ether	2CO + 4H_2_ = CH_3_OCH_3_ + H_2_O

**Table 2:** Methanol yield (t/day) guaranteed by various productive processes, depending on the feedstock.

Productive Process	Feedstock	CH_3_OH Yield (t/Day)
BASF	Syngas	7.9 × 10^−2^
Dupont	Syngas	1.1·× 10^−1^
Haldor Topsoe	Syngas	2.4·× 10^3^
ICI	Carbonaceous	2.5·× 10^3^

**Table 3:** Performance of methanol steam reforming (MSR) reaction in membrane reactors (MRs): Pd/Pd-alloy and not Pd-based membranes application.

Membranes	Membrane Preparation	Metallic Layer (μm)	Catalyst	H_2_O/CH_3_OH	T (°C)	p (bar)	Conv. (%)	H_2_ Recovery (%)	H_2_ Purity (%)	Ref.
Pd-Ag, Pd-Ru	Magnetron sputtering	60, 12	Ru/Rh/ZrO_2_	1/1	300	-	-	38, 18	≈100	[90]
Pd-Ag	-	6	Cu/ZnO/Al_2_O_3_	1/1	300	2	98	64	-	[91]
Pd/Al_2_O_3_	ELP	7	CuO/ZnO/Al_2_O_3_	2.5/1	330	2.5	85	>40	≈100	[92]
Pd-Ag/TiO_2_-Al_2_O_3_	ELP	-	Ru-Al_2_O_3_	4.5/1	550	1.3	65	-	≈72	[93]
Pd/PSS	ELP	20	Cu/ZnO/Al_2_O_3_	1.2/1	350	6	≈95	97	99.9	[94]
Pd-Ag/α-Al_2_O_3_	ELP	~4	CuO/ZnO/Al_2_O_3_	1/1	250	3 10	100	45 95	≈100	[95]
Pd-Ag/PSS	ELP	20–25	CuO/ZnO/Al_2_O_3_	1.2/1	240	10	36.1	18	-	[96]
Pd/PSS	ELP	~20–25	Cu-based	1.2/1	350	-	99	-	≈100	[97]
Pd-Ag	Cold-rolling	50	CuO/Al_2_O_3_/ZnO MgO	3/1	300	3	-	80	≈100	[98]
Pd-Ru-In	-	200	Cu/ZnO/Al_2_O_3_	1.2/1	200	7	≈90	≈24	≈100	[99]
Pd-Cu	-	25	Cu-Zn based	-	300	10	>90	≈38	≈100	[100]
Carbon molecular sieve	Pyrolysis	-	CuO/ZnO/Al_2_O_3_	4/1	200	1	≈95	≈84	-	[101]
Carbon supported	Pyrolysis	-	CuO/Al_2_O_3_/ZnO MgO	3/1	250	2	55	-	≈80	[102,103]
Carbon supported	-	-	Cu/ZnO/Al_2_O_3_	1.5/1	250	2	≈99	-	97	[104]
SiO_2_/γ-Al_2_O_3_/Pt-SiO_2_/PSS	Soaking-rolling	-	Cu-Zn based	1.3/1	230	-	100	~10	-	[105]
SiO_2_/γ-Al_2_O_3_	Soaking-rolling	-	Cu-Zn based	3/1	260	-	42	5	98	[106]

## List of Acronyms

ACL	Anode Catalyst Layer
AGDL	Anode Gas Diffusion Layer
AMR	Autothermal Methane Reforming
BASF	BadischeAnilin und Soda Fabrik
CR	Conventional Reactor
DME	DiMethyl Ether
DMFC	Direct Methanol Fuel Cell
ELP	Electroless Plating
GHG	Greenhouse Gases
HT	High Temperature
ICI	Imperial Chemical Industries
MD	Methanol Decomposition
MR	Membrane Reactor
MSR	Methanol Steam Reforming
MTBE	Methyl Tertiary Butyl Ether
NCCC	National Carbon Capture Center
PEM	Proton Exchange Membrane
PMR	Photocatalytic Membrane Reactor
POM	Partial Oxidation of Methanol
SMR	Steam Methane Reforming
WGS	Water-Gas Shift
